# Utility of lay and clinical narratives for transparent autism diagnosis using BioBERT deep learning

**DOI:** 10.3389/fdgth.2026.1804334

**Published:** 2026-07-17

**Authors:** Gondy Leroy, Himanshu Nimbarte, Madhuri Sai Kandula, Prosanta Barai, Sumi Lee, Nell Maltman, Sydney Rice

**Affiliations:** 1 Department of Management Information Systems, Eller College of Management, University of Arizona, Tucson, AZ, United States; 2 Department of Linguistics, College of Social and Behavioral Sciences, University of Arizona, Tucson, AZ, United States; 3 Department of Speech, Language and Hearing, College of Science, University of Arizona, Tucson, AZ, United States; 4 Department of Pediatrics, College of Medicine, University of Arizona, Tucson, AZ, United States

**Keywords:** autism spectrum disorder, bioBERT, clinical narrative, DSM-5, lay language, machine learning, topic analysis

## Abstract

**Introduction:**

Early autism diagnosis remains challenging due to reliance on clinical observation and limited specialist availability. Addressing these barriers through automated diagnostic labeling and the integration of parental input may help mitigate the problem.

**Methods:**

We trained a BioBERT machine learning model to label individual autism behavioral descriptions using the seven DSM-5 diagnostic criteria (A1-A3, B1-B4). This approach offers transparent clinical decision-making by providing detailed diagnostic information for individual behaviors and avoiding final case-level black-box decisions. We evaluated the model's performance on labeling lay (*N* = 35,971) and clinical (*N* = 145,603) behavior descriptions, as well as its transferability between the two. In addition, we compared the data sources by evaluating the diagnostic utility of lay and clinical examples across four dimensions, and of AI-generated summaries across two dimensions.

**Results:**

We found that BioBERT can label both types of input, although it achieved higher precision (69%) on clinical descriptions and higher recall (83%) on lay descriptions. Sample size did not explain differences in performance. Transferring models from one data type to another results in a performance drop. Overall, training first on clinical data yielded the best-performing diagnostic models. When evaluating the examples from both data sources, the results show similar scores for the Utility, Specificity, Clinical Relevance, and Impact on Daily Life dimensions, and the cosine similarity analysis revealed substantial overlap (0.42) in vocabulary between the two. The utility of examples for A diagnostic behaviors was generally scored higher than that for B diagnostic behaviors. AI-generated summary scores showed a similar pattern between A and B examples but they were only moderately representative of these examples.

**Discussion:**

These results demonstrate that lay behavioral descriptions can provide diagnostically valuable information comparable to clinical observations, although they are not readily summarized by AI. The integration of lay information into the diagnostic workflows could accelerate autism diagnosis without compromising clinical utility.

## 1 Introduction

Autism spectrum disorder (ASD) is a neurodevelopmental condition affecting a growing number of children (1). Early intervention is proving most effective for improving long-term outcomes (2) and delays in diagnosis hinder timely intervention and reduce the impact of therapeutic support. Despite advances in awareness, early diagnosis remains a significant challenge. Current methods rely on clinical, often in-person, observation of behavior, a process that is time-consuming, resource-intensive, and limited by practical obstacles. In addition, clinicians must be knowledgeable to navigate overlapping symptoms with other neurodevelopmental disorders, variability across the autism spectrum, and age-dependent behavioral expressions. Digital health approaches can facilitate and enhance decision-making (3), and have been shown to be effective for a variety of mental health conditions (4).

New research approaches aim to develop diagnostic measures that do not rely on observation by identifying biological or physiological markers associated with ASD. Ideally, differences can be detected even before behavioral differences are noted (5). Several different neuroimaging techniques (e.g., MRI, EEG) have been tested to detect structural or functional differences indicative of ASD. For example, a review of twelve clinical trials has shown the potential of associating brain lesions with psychiatric disorders (6). While these tools show promise in capturing differences before behavioral symptoms emerge, small sample sizes, lack of standardization, and high costs limit scalability (5). Genetic and molecular markers are also being increasingly explored. Similarly, these approaches face significant obstacles in diagnosing, due to the complexity caused by genetic heterogeneity: no single gene variant explains all cases, and not all relevant genes are affected in all patients (7).

Machine learning (ML) has been applied to a variety of data, such as eye movements and behavioral assessments, for which large datasets can be created. Eye-movement analysis, with or without machine learning, was found to enable tracking social engagement in children between 16 and 30 months old and had increased value when combined with facial expressions, especially with adults (8). Several studies leveraged traditional and deep machine learning with behavioral assessments and eye-tracking data (9–11), or facial expressions (12). Some models have shown promising results in early detection. However, the results are not uniformly positive. A review of nine machine learning approaches (13) showed that identifying young children with ASD based on survey screening instruments was not very successful, using acoustic information was better, while using brain information had reduced success with older children.

Many of these new methods are promising, but they require controlled environments or calibration, thereby limiting their real-world applicability. Acquiring the necessary datasets is another significant obstacle (14). Furthermore, most algorithms are binary (autism or not) black-box algorithms that provide no rationale and require blind trust in the algorithm. This approach is misaligned with diagnosing many mental health conditions, and especially autism, which is expressed with a variety of different phenotypes and with different severities. While these innovations highlight progress, no unifying framework has emerged for clinical use. Cost, accessibility, and the need for expert interpretation further delay widespread adoption.

Clinical observation remains the primary approach for autism diagnosis. This also has limitations, including professional biases and caregiver burden. Clinical observation aims to be objective, but it may miss signs present in everyday life that are culturally specific (e.g., eye contact may be considered rude in some cultures) and indicative of ASD. Parental input has largely been gathered using structured surveys, such as the M-CHAT-R, and through structured tools, such as the ADOS-2 or the CARS-2, to streamline screening. Future tools would benefit from harmonizing emerging biomarkers with behavioral data while prioritizing accessibility. Low-cost, parent-reported tools (e.g., digital platforms that capture video or text) could supplement clinical visits, thereby reducing delays.

We believe our work is among the first to compare lay and clinical descriptions as diagnostically relevant input. Our use of free-text narratives contrasts with the traditional structured questionnaires for parental input, making this comparison possible. We use machine learning (ML), i.e., BioBERT, to label descriptions of children's behaviors using DSM-5 diagnostic criteria. Although labeling relies on opaque deep learning, by labeling behaviors, our approach is transparent, and the diagnostic rationale is clear, which is in contrast to most other deep learning approaches focusing on a final diagnosis (3). Furthermore, the use of parental input may improve access to services in different economic settings, a desired feature that is often ignored (15).

In prior work, we have shown that even with imperfect ML labeling of individual behaviors, sensitivity and specificity for diagnosing an individual (i.e., case level) are comparable to those of clinical expert decisions (16), allowing us to focus on individual criteria in this work. We conducted a dual analysis. First, we evaluated ML performance in automated labeling of lay and clinical descriptions and ML transfer between the two data types. Second, we provided a clinical expert analysis of lay and clinical examples, as well as AI summaries of these examples. We found that ML is more precise with clinical descriptions, but labels more behaviors correctly with lay descriptions. Models that need to label both types of descriptions are best trained on clinical data first. There is substantial similarity in lay and clinical descriptions, with the highest similarity for A2 and the lowest for B2 examples. The lay and clinical examples were considered to have similar diagnostic utility, with A-examples being generally scored higher than B-examples. AI-generated summaries of examples were more representative of lay than of clinical examples, but need to improve to stand in for the examples themselves.

## 2 Materials and methods

### 2.1 Datasets

To gather clinical behavioral descriptions, we extracted clinical note records from the Centers for Disease Control and Prevention (CDC) Autism and Developmental Disabilities Monitoring (ADDM) Network, which contain records for 8- and 4-year-old children living in Maricopa County (Arizona) (8), collected in 2016. We added records from the University of Arizona Clinical Data Warehouse (CDW) comprising EHR with ICD10 diagnostic codes related to autism (F84.0 Childhood Autism, F84.5 Asperger Syndrome, F84.1 Atypical Autism, F84.8 Other pervasive developmental disorders, F84.9 Pervasive developmental disorders, unspecified, F84.4 Overactive disorder associated with mental retardation and stereotyped movements, F84.2 Rett's Syndrome, F84.3 Childhood Disintegrative Disorder) and one ICD10 screening code (Z13.41—encounter for autism screening. Our clinical team reviewed individual sentences in the clinical language datasets and labeled them with DSM-5 criteria (the process is described below).

To collect lay behavioral descriptions, we developed a 29-item survey aligned with DSM-5 criteria but phrased in lay language. Table 1 provides examples of criteria and associated questions. We collected responses by distributing a Qualtrics survey within our clinical network This data was collected as part of a larger dataset in which we focused on improving the quality of crowdsourced data. The data were collected over several weeks. Initial results indicated that many lay responses were unusable; therefore, we developed quality-control measures to ensure that no responses were copied and pasted or sourced from existing websites. These controls were largely automated (17),. Then we removed nonsensical answers, such as song lyrics. Our clinical collaborators shared the link with their networks. Respondents were paid a $20 Amazon gift card for completing at least 25 questions. We invited respondents who self-identify as being caregivers of children with autism or who self-identify as being diagnosed themselves. The majority of the respondents were male (64% male and 35.4% female) and reported on people with autism, the majority of whom were male (67.1% male and 32.4% female). The respondents were mostly reporting on behalf of others (42.3% were parents or guardians, spouses or partners, 43.4% friend, and 9% were educators). They reported in 97.9% on someone diagnosed with autism (diagnosis was not verified by the investigator).

**Table 1:** Example data collection questions.

DSM criteria	Example survey question
A1 Deficits in social-emotional reciprocity, ranging, for example, from abnormal social approach and failure of normal back-and-forth conversation, to reduced sharing of interests, emotions, or affect, to failure to initiate or respond to social interactions.	Does this person use words or answers that you would not expect in a typical interaction (e.g., changing the subject, not answering a question)? Please give a concrete example.
	Does this person not use sounds (e.g., haha, hmm,) as you expect in a typical interaction? Please give a concrete example.
A2 Deficits in nonverbal communicative behaviors used for social interaction, ranging, for example, from poorly integrated verbal and nonverbal communication; to abnormalities in eye contact and body language or deficits in understanding and use of gestures; to a total lack of facial expressions and nonverbal communication.	Does this person not use gestures (e.g., use of hands, nods) in a typical manner? Please give a concrete example.
	Does this person not behave in a typical social manner (e.g., making eye contact, turning body toward the person speaking)? Please give a concrete example.
A3 Deficits in developing, maintaining, and understanding relationships, ranging, for example, from difficulties adjusting behavior to suit various social contexts; to difficulties in sharing imaginative play or in making friends; to absence of interest in peers.	Does this person not have any long-term, high-quality relationships with family, friends, or colleagues? Please give a concrete example.
	Does this person not adjust behaviors when the surroundings or social context change (e.g., on the playground, in clubs)? Please give a concrete example.
B1 Stereotyped or repetitive motor movements, use of objects, or speech (e.g., simple motor stereotypies, lining up toys or flipping objects, echolalia, idiosyncratic phrases).	Does this person not use their hands and arms in a typical manner? Please give a concrete example.
	Does this person not walk in a typical manner (e.g., walk on their toes or be stiff when moving)? Please give a concrete example.
B2 Insistence on sameness, inflexible adherence to routines, or ritualized patterns or verbal nonverbal behavior (e.g., extreme distress at small changes, difficulties with transitions, rigid thinking patterns, greeting rituals, need to take same route or eat food every day).	Does this person stick to routines or patterns of behaviors more than typical (e.g., they do exactly the same thing every day)? Please give a concrete example.
	Does this person become upset or react differently when you change plans (e.g., planning to go to the grocery store and then the bank but switching the order is upsetting)? Please give a concrete example.
B3 Highly restricted, fixated interests that are abnormal in intensity or focus (e.g, strong attachment to or preoccupation with unusual objects, excessively circumscribed or perseverative interest).	Does this person focus excessively or focus overly long on an area of interest? Please give a concrete example.
	Does this person focus excessively or focus overly long on any objects? Please give a concrete example.
B4 Hyper- or hyporeactivity to sensory input or unusual interests in sensory aspects of the environment (e.g., apparent indifference to pain/temperature, adverse response to specific sounds or textures, excessive smelling or touching of objects, visual fascination with lights or movement).	Is this person extremely sensitive or attracted to certain types of touch (e.g, licking non-food items or cannot wear shirts with tags)? Please give a concrete example.
	Is the person insensitive to certain types of touch (e.g., don't notice touching their arm)? Please give a concrete example.

The clinical labeling process for both lay and clinical examples was the same. All text was presented sentence by sentence to human reviewers who indicated if a sentence was relevant or not. Those sentences that were relevant were labeled with DSM criteria. Two expert ASD clinicians labeled individual behaviors (e.g., repetitive motions) using DSM-5 criteria (A1–3, B1–4). They were trained to ensure inter-rater reliability ≥80% for ASD case classification and ≥90% for criterion-specific labeling, in accordance with accepted CDC ADDM Network benchmarking standards.

The final data contained 35, 971 and 145,603 items, of which 16,885 (46.9%) lay descriptions and 9,514 (6.5%) clinical descriptions received a DSM-5 label (Table 2). The number of lay descriptions receiving a label is naturally higher, since this dataset is derived from the ASD-focused survey. The percentage of labeled items in the clinical data is lower, since the starting point for this dataset was the clinical notes in the records, which naturally contain unrelated information. Among labeled examples, criterion B1 received the most examples for lay descriptions (20.5%), and criterion A1 received the most examples for clinical descriptions (22.5%). The smallest set of examples was for the A1 criterion for lay descriptions (9.9%) and for the B3 criterion for clinical descriptions (5.0%).

**Table 2:** Data labeled with ASD DSM5 criteria.

Counts	Data for machine learning		Data subset for clustering	
	Lay data:	Clinical data	Lay data: qualtrics	Clinical data: ADDM
Total example count	35,971	145,603		
No labels assigned	20,486	137,191		
Labels assigned	16,885	9,514	13,297	4,836
Criterion	*N* (%)			
A1	1,669 (10)	2,136 (22)	1,126 (8)	1,167 (24)
A2	1,732 (10)	1,323 (14)	1,297 (10)	611 (13)
A3	2,781 (16)	1,436 (15)	2,296 (17)	738 (15)
B1	3,461 (20)	1,522 (16)	2,951 (22)	692 (14)
B2	1,810 (11)	895 (9)	1,327 (10)	485 (10)
B3	2,219 (13)	476 (5)	1,794 (13)	226 (5)
B4	3,213 (19)	1,726 (18)	2,506 (19)	917 (19)

The complete dataset was used to train and test machine learning algorithms for diagnostic labeling, ensuring comprehensive coverage of diverse linguistic expressions and stylistic variations. For the content analysis, we clustered the examples to make the evaluation manageable. We restricted the dataset to the Qualtrics survey (lay) and ADDM records (clinical) subsets, since these sources are more specifically oriented toward ASD-related content.

### 2.2 Machine learning models

For both data sets, we used a BioBERT (Version 1.2) model: a domain-specific model pre-trained on medical corpora. We have shown in prior work that the BioBERT model performed better than other deep learning models, as well as ensembles of such models (16). Supplementary Appendix A contains the model parameters for both.

### 2.3 Criterion labeling analysis

We evaluated the BioBERT model using 10-fold cross-validation and report the average score for precision, recall (i.e., sensitivity or True Positive Rate), and F1 scores (i.e., harmonic mean representing the balance between the two):

$$\mathrm{Prcision}=\frac{TP}{TP+FP}$$

$$\mathrm{Recall}=\frac{TP}{TP+FN}$$

$$F1=2*\frac{\mathrm{Precision}*\mathrm{Recall}}{\mathrm{Precision}+\mathrm{Recall}}$$

with TP = True Positives, TN = True Negatives, P = False Positives, and FN = False Negatives.

To evaluate model transfer, we select the fold with the best-performing model and its associated dataset for both the lay and clinical data. The best BioBERT model is then applied to the data from the best fold in the other dataset. E.g., the best model for lay descriptions is selected and applied to the data from the best fold for clinical descriptions. This approach allows a clear evaluation of model transfer. Note that with final deployment, models would be retrained on 100% of the data.

We sequentially trained on the two datasets and on the combined dataset. The first scenario represents a common situation in which a software package is acquired and further tuned using in-house data. The second scenario represents the opportunity to train a model on all combined data from the start.

### 2.4 Criterion content analysis

To evaluate the quality of lay and clinical examples for diagnosing, two clinical experts evaluated representative examples for each criterion. However, because the datasets were too large to evaluate all examples, we clustered them and presented an AI-generated summary, along with five original examples from each cluster. We chose this method over random selection from all examples to facilitate broader coverage, since similar examples are clustered and shown only once to the evaluator. Furthermore, this approach enables us to evaluate generative AI for summarizing examples.

To cluster the examples, all examples were first represented as vectors generated by a lightweight, domain-agnostic sentence-transformer model (all-MiniLM-L6-v2). Cosine similarity between examples was used to compare vectors (via L2-normalised sentence embeddings). Using this representation, we compared K-Means, Agglomerative Clustering, and HDBSCAN with 5, 10, and 15 clusters for all descriptions associated with a certain criterion. We used the clustering algorithms internal metrics to choose the best models: Silhouette, Calinski–Harabasz, and Davies–Bouldin. Each raw score was normalized and converted to a Z-score so that higher is better (Davies–Bouldin Z-scores were sign-inverted) and combined into composite quality measures. We used the mean of the three scores (weighted equally) to select the clusters.

In addition to the examples, we generated concise, clinician-facing titles to summarize each cluster. We used a locally hosted Llama-3-8B-Instruct model (8B parameters) deployed on our high-performance computing (HPC) environment. For each cluster, all de-duplicated examples were concatenated and passed to the model with a structured prompt instructing the model to act as an expert pediatrician and produce a single-sentence title (≤20 words), in plain clinical English, referencing at least one prominent observed behavior or test finding, without names, dates, identifiers, or invented information. The prompt is provided in Supplementary Appendix B.

Per diagnostic criterion (A1, A2, etc.), the clusters (title and five examples) from lay and clinical datasets were randomized and shown without information about the underlying algorithm to our experts. They evaluated these examples using a 4-point scale for their usefulness in diagnosing:

- Utility: The examples are related to each criterion.
- Specificity: The examples represent autism (not other neurodevelopmental conditions).
- Clinical Representativeness: The examples are likely to be witnessed by a clinician.
- Impact on Daily Life: The examples describe obstacles encountered in daily life.

The AI-generated titles summarizing the examples were also evaluated by our experts for conciseness (Concise) and representativeness (Representative) using a 4-point scale (1: Do not agree at all; 2: Somewhat Agree; 3: Agree; 4: Completely Agree).

## 3 Results

### 3.1 Criterion labeling (machine learning)

Table 3 (Top Half: Initial Training) shows the results for ML criterion labeling for lay and clinical text. When examining average performance across all labels, we find that the ML performance on clinical descriptions achieved 69% precision and 54% recall (F1 score: 0.64). For the lay text, average precision was 50% with 83% recall (F1 score: 0.54). When the two data types were combined, the model's performance showed lower precision, 33%, but higher recall, 83%, (F1 score: 0.36).

**Table 3:** Accuracy of labels at criterion level for Lay and clinical text data.

Initial training									
	Lay text			Clinical text			Lay + clinical text		
Label	Prec.	Recall	F1	Prec.	Recall	F1	Prec.	Recall	F1
A1	0.99	0.22	0.36	0.68	0.55	0.61	0.99	0.28	0.43
A2	0.33	0.88	0.48	0.70	0.71	0.70	0.12	0.91	0.21
A3	0.42	0.97	0.59	0.69	0.43	0.53	0.17	0.92	0.28
B1	0.51	0.95	0.66	0.70	0.66	0.68	0.26	0.94	0.41
B2	0.37	0.87	0.52	0.74	0.53	0.62	0.23	0.91	0.37
B3	0.37	0.94	0.53	0.67	0.29	0.40	0.19	0.90	0.31
B4	0.47	0.99	0.64	0.66	0.60	0.63	0.32	0.98	0.48
Macro average	0.50	0.83	0.54	0.69	0.54	0.60	0.33	0.83	0.36
Best Model Lay (5)	0.50	0.83	0.62	0.35	0.55	0.43	0.49	0.82	0.61
Best Model Clinical (7)	0.42	0.34	0.38	0.73	0.59	0.65	0.74	0.76	0.75
Best Model Mixed (7)	0.48	0.74	0.58	0.36	0.54	0.43	0.47	0.82	0.60

Further tuning									
	Clinical text			Lay text					
	Prec.	Recall	F1	Prec.	Recall	F1			
A1	0.67	0.47	0.55	0.75	0.23	0.35			
A2	0.75	0.67	0.71	0.71	0.70	0.71			
A3	0.71	0.39	0.50	0.58	0.70	0.63			
B1	0.70	0.61	0.65	0.67	0.85	0.75			
B2	0.72	0.50	0.59	0.48	0.57	0.52			
B3	0.61	0.30	0.40	0.53	0.62	0.57			
B4	0.65	0.55	0.59	0.57	0.76	0.65			
**Macro average**	**0.69**	**0.50**	**0.57**	**0.61**	**0.63**	**0.60**			

When focusing on individual DSM-5 criteria, there are particularly clear trade-offs between precision and recall in lay descriptions. For example, for the A1 label, precision is nearly perfect (0.99), whereas recall is very low (0.22). The reverse is seen for B4, where precision is low (0.44) but recall is nearly perfect (0.99). For the clinical descriptions, the trade-offs are less extreme, except for B3, which achieves 67% precision and 29% recall. When the data is mixed, the models achieve high recall but at the price of low precision. For lay descriptions, the most balanced results are obtained for the B1 criterion, with 51% precision and 95% recall (F1 score: 0.66). A1 had the highest precision (99%), and B4 had the highest recall (99%). For clinical descriptions, the most balanced results were found for the A2 criterion with 70% precision and 70% recall (and 0.70 F1-score). The highest precision was 74% for criterion B2, and the highest recall was 71% for criterion A2. For the mixed data, the most balanced data were obtained for criterion B4, although precision and recall remained low at 32% and 98%, respectively (F-score: 0.48). In this mixed dataset, criterion A1 has the highest precision, 99%, and criterion B4 has the highest recall, 98%.

Table 3 (Bottom Half: Further Tuning) also shows the results for the transfer learning. The best lay model was found with fold 5. The best clinical model was found for fold 7. Without further tuning, the best lay model (from fold 5), shows a decrease in precision of 8% (from 50% to 42%) and a decrease in recall of 49% (from 83% to 34%) when applied to clinical descriptions. We observe similar drops in performance when the clinical model is applied to lay text.

We then proceeded with the best initial model for further tuning and found improved performance. For the lay model further tuned and tested on clinical text, we find an increase in precision from 50% to 69% but a drop in recall from 83% to 50%. When tuning and testing the clinical model on lay text, precision drops from 69% to 61%, and recall increases from 54% to 63%.

Our datasets are imbalanced, with many more negative than positive examples, especially in the clinical dataset. In such settings, models are often biased toward predicting the negative class and achieve higher accuracy on these datasets. However, we compare macro precision and recall across all diagnostic labels. The differences between datasets are modest: in the lay dataset, we obtain 50% precision and 83% recall, whereas in the clinical dataset, we obtain 54% precision and 69% recall, corresponding to *F*-values of 0.54 and 0.60, respectively. To better understand model behavior under this imbalance, we conducted a follow-up analysis focusing on the positive examples. We calculated two-tailed Pearson correlations between precision/recall and the number of examples for each label in each dataset (Supplementary Appendix C: Table 6). We did not observe any significant correlations, suggesting that performance differences were not driven by label frequency.

### 3.2 Criterion content analysis for diagnosis

#### 3.2.1 Expert evaluations

First, we calculated the agreement between the two experts using two-tailed Pearson's r correlation analysis. We chose correlations over Cohen's kappa because the ratings are numeric scores, and we are primarily interested in whether the reviewers scored in the same direction across the evaluation dimensions. We calculated correlations for the six dimensions and for lay and clinical data. With a Bonferroni correction for twelve comparisons, the significance threshold is reduced to.004. For the clinical data, the scores are significantly and positively correlated for all dimensions. For the lay data the scores are positively correlated for four of the six dimensions. This indicates broad agreement, especially for clinical data, although somewhat lower for the lay data. The detailed correlation coefficients for each dimension are reported in Supplementary Appendix E.

#### 3.2.2 Similarity between lay and clinical descriptions

The cosine similarity analysis between lay and clinical descriptions of autism behaviors revealed variable degrees of similarity (Table 4). Overall, the similarity between lay and clinical text was 0.42 for the labeled examples and 0.25 for text that was not labeled. This reflects that labeled text is more focused (as intended).

The highest similarity between the two sources was observed for A2 behaviors (0.59), followed by B1 (0.50) and A1 (0.46), indicating substantial overlap in vocabulary and content between professional and non-professional descriptions for these behaviors. Moderate similarity was found for behaviors B4 (0.41), B3 (0.39), and A3 (0.37), suggesting partial alignment in terminology. Behavior B2 showed the lowest similarity (0.36).

#### 3.2.3 Quality of lay and clinical descriptions and AI-summary titles

After comparing different clustering algorithms, the best results were achieved with the K-Means algorithm with 15 clusters for the lay text and 5 clusters for the clinical text. The difference in the number of clusters reflects that more different topics were present in the lay text than in the clinical text.

**Figure 1:**
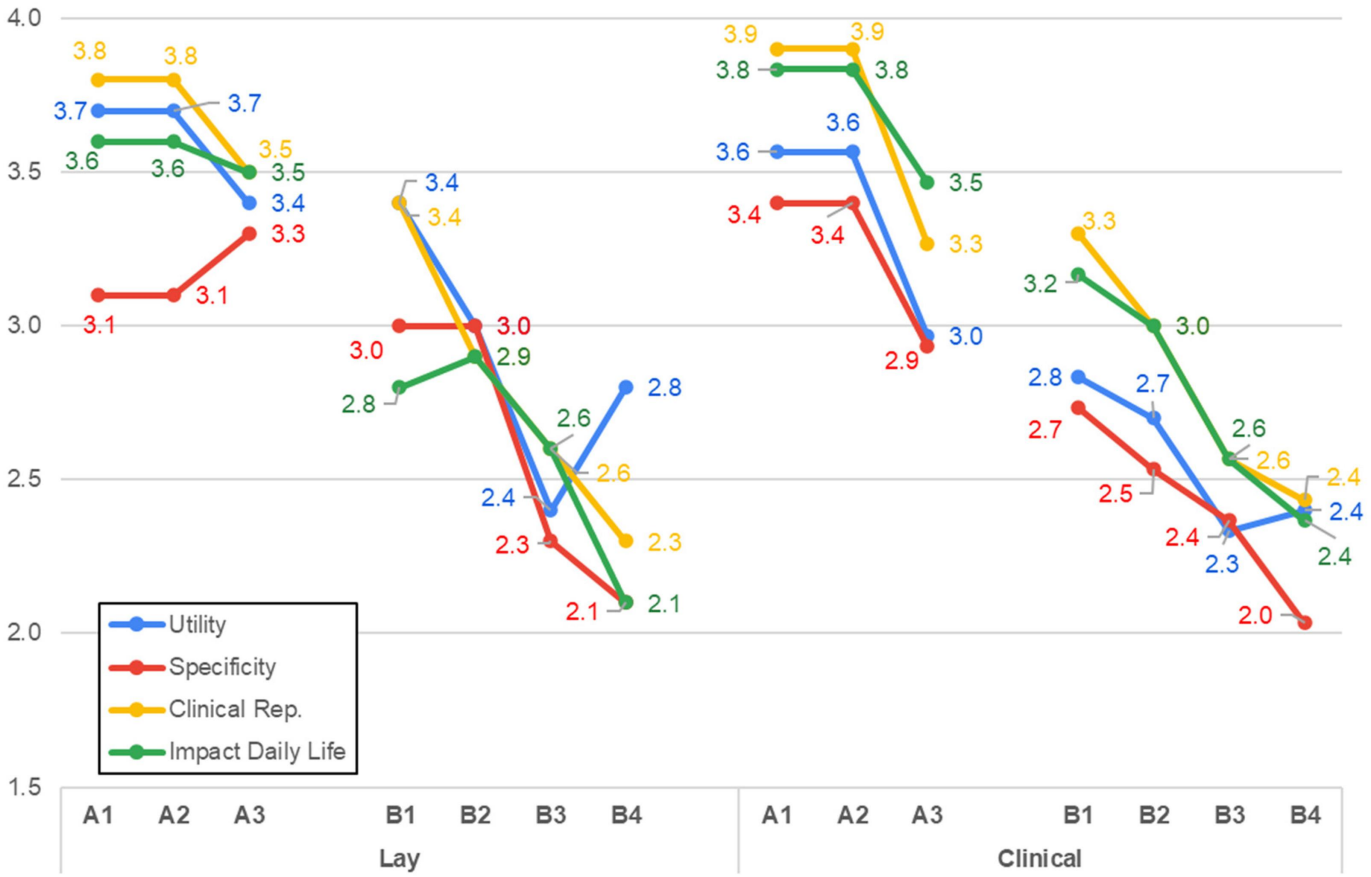
Average lay (*N* = 15) and clinical (*N* = 5) scores for A and B criteria examples.

In Figure 1, we show the expert scores for the lay (left pane) and clinical examples (right panel). (Details and statistical results are provided in Supplementary Appendix D). Overall, lay and clinical examples received similar scores. The A1 and A2 examples received the highest scores on all dimensions for both lay and clinical texts. The B3 and B4 examples received lower scores on all scored dimensions.

Across individual dimensions, the utility of lay examples is slightly higher (3.2 for lay vs. 2.9 for clinical), but this difference is not statistically significant. The average scores for the Specificity and for Clinical Representativeness are identical (2.8 and 3.2, respectively) and only slightly different (not statistically significant) for Impact on Daily Life (3.0 and 3.2, respectively).

While most evaluations follow the same pattern, there are small differences. For example, the utility of lay examples is higher than that of clinical examples for A3 and B4 examples. The specificity for A3 is also higher for lay compared to clinical examples.

**Figure 2:**
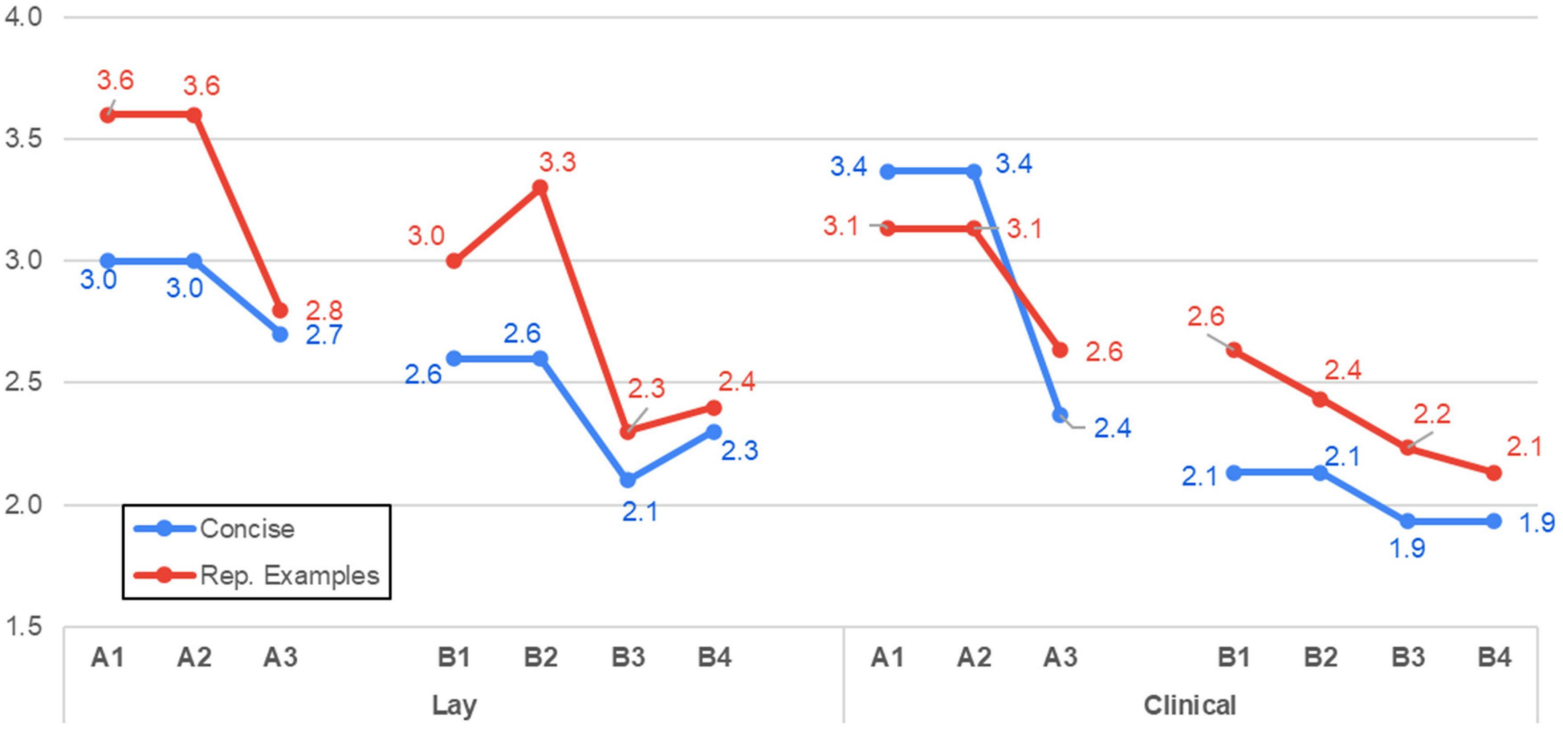
Average lay and clinical scores for AI-generated summaries.

In Figure 2, we present the expert scores for the AI-generated summary (Details and statistical results are also in Supplementary Appendix D). In general, the summary values follow the same patterns as the examples and are highest for summaries of the A1 and A2 criteria across both lay and clinical examples. The conciseness of the summary is higher in lay (2.6) than in clinical (2.5) examples; however, this difference is not statistically significant. The representativeness of the summary was also higher for lay (3.0) than for clinical (2.6) examples, a significant difference [F(1,138) = 5.824, *p* = 0.017].

The average scores (across all dimensions) for the AI-generated summaries and examples correlate very highly (*r* = 0.907, *p* < .001). However, AI-generated summaries were only moderately representative of the underlying examples and would need improvement before they could serve as a stand-in for individual examples.

Table 5 shows several examples of clusters and their scores for criterion A1 and B1 for both the lay and clinical text.

**Table 4:** Cosine similarity between criteria for Lay and clinical text.

Label	Cosine Similarity
A1	0.46
A2	0.59
A3	0.37
B1	0.5
B2	0.36
B3	0.39
B4	0.41
No Label	0.25
Average	0.42

**Table 5:** Examples in clusters and their AI generated summaries.

Criterion	Origin	Examples	Examples Score (Avg of Metrics)	AI-Generated Summary	Summary Score (Avg of Metrics)
A1	Lay	When asked about something, he produces a particular sound to show that he does not like the question or the conversation.	3.88	Child exhibits inconsistent verbal communication responses and unusual sounds.	3.50
		She may appear not to hear what you say to her,not respond to her name or appear to any attempts you make to communicate; she often understands language literally and have difficulty with understanding and using the natural rythm in conversation			
		sometimes ask him if he wants to go to bed and he starts to narrate some other experience he had in the past not giving an answer to what was asked			
		my friend always randomly uses words that are offensive in the middle of a conversation hence makes it hard for someone to understand him			
		when answering he may give answers that are not related to the question that one asked.			
A1	Clinical	His scores on the Pragmatic Language Profile revealed inadequate pragmatic skills across raters in the areas of rituals and conversational skills and asking for/giving/responding to information.	4.00	Moderate to severe pragmatic language impairments, including difficulties with rituals, conversations, and turn-taking.	3.25
		Analysis of Speech and Language Results He demonstrates excellent skills in the area of Comprehension/Semantic skills, he is able to follow directions He demonstrates average skills in the area of Verbal Expression/Form Finally, in the area of Social Pragmatic Language, he demonstrates reduced skills when compared to others students his age.			
		In addition the documentation states that he presents with overall delays in the areas of receptive, expressive language and pragmatic language.			
		Speech/language summary: Overall, he demonstrates average receptive and expressive language skills for his age, yet he does demonstrate a delay in pragmatic language skills.			
		His difficulties with pragmatic language and social skills behavior influence his social and academic communication and performance.			
B1	Lay	walks on his toes	3.38	The child exhibits an unusual gait pattern, often walking on the balls of his/her feet or toes.	3.00
		Walking on toes			
		walks on toes			
		Walking with his toes			
		walk on toes			
B1	Clinical	He also repeats words/phrases over and over.	3.67	Echolalia and repetitive speech patterns, including word and phrase repetition, observed in various contexts.	3.00
		He was heard to repeat the word water several times.			
		He often repeats the question.			
		Repeats words out of context [i.e., repeats words heard at an earlier time].			
		At times, he was observed responding to questions by repeating part of the question.			

## 4 Discussion

Overall, machine learning models performed better on clinical text. While the averages do not show a large difference, the lay descriptions exhibit much greater variance. We believe this difference in model performance is due to clinical text being more standardized, possibly as a result of clinicians' training and practice, and potential copy and paste in clinical records, compared to descriptions by lay people. When two distinct data types must be labeled by a single model, our transfer-learning analysis showed that it is best to first train the model on clinical text and then fine-tune it on lay text. This contrasts with our earlier work, in which we trained on two similar clinical datasets (18), and training on mixed data yielded slightly better performance.

While precision and recall values for individual criteria may seem low, our prior work showed that when combined into a diagnostic suggestion, sensitivity and specificity are comparable to or higher than those of human experts (16). This is because a diagnosis does not require every behavior to be labeled correctly. For example, if 10 sentences are labeled with A1 and only a few were correctly labeled, precision may be low, but this is sufficient to support the diagnosis, since only one example behavior would be needed for A1. Similarly, even with low recall, there may still be ample evidence to support an autism diagnosis, and not every behavior requires extensive labeling. We do not calculate sensitivity and specificity in this work because no verified diagnoses were possible from the lay text.

The quality of the examples was high and comparable for lay and clinical text for utility in diagnosing, even though it was somewhat lower for B criteria examples. We believe this demonstrates the value of lay descriptions in the diagnostic process, reflecting a promising alternative route to diagnosing children that is less restricted by geographical burdens and time constraints. Behavioral descriptions provided by parents have promise for the development of diagnostic tools. While the survey questions were anchored in DSM-5 criteria to focus on clinically relevant behavior, respondents provided open-ended descriptions in their own language, which allowed variation in content and phrases and reduced direct cueing toward a fixed diagnostic answer. These narratives are inherently closer to real-world behavior.

The AI-generated summary titles were only moderately representative of the underlying examples. This shows that human evaluation is necessary for AI-generated text. It also may reflect the difficulty of summarizing a wide variety of examples such as those of the B criteria and the need for more sophisticated prompts.

Our goal in the transfer learning analysis was to demonstrate a proof of concept for whether representations learned in one setting could be reused in the other. Our rationale was that by choosing the best model, we would show the largest potential impact. Future work should include a more exhaustive evaluation using a dedicated held-out set and applying the completely retrained model (all folds). In addition, we aim to develop models that can process multiple versions of input. This may require larger models and more training examples. However, as shown in our correlation analysis, sample size is not the primary driver of performance, and the language type or the diversity of examples may be partially responsible.

Furthermore, a starting point with mixed datasets resulted in lower performance. This may be driven by having two datasets that differed in source data and vocabulary, and therefore likely reflected different underlying distributions; when combined, this may have increased domain variability and diluted the predictive signal, and through attention dilution in BERT. In future work, larger dataset-specific samples or more harmonized data collection may help determine whether performance improves when the model is trained on more consistent data for each source.

Finally, we focus on identifying clinical factors crucial to an autism diagnosis, using a BioBERT-based model to capture complex patterns in the data. While this BioBERT model is opaque in its labeling, the final diagnostic decision remains transparent and is ultimately made by clinicians rather than by the model itself. This stands in contrast to approaches that use machine learning to produce the final diagnostic decision and apply *post hoc* explanation methods (for example, SHAP or LIME) to interpret the contribution of individual variables for autism and other diagnoses (18–21). Both strategies offer complementary strengths: fully automated, *post hoc*–explained models can optimize predictive scalability, whereas our design prioritizes explicit clinical control at the point of diagnosis.

## 5 Conclusion

To our knowledge, we are the first to gather and compare lay and clinical examples of children's behaviors that align with DSM diagnostic criteria. Our ML algorithms show that both can be used in the diagnostic process, although clinical examples are slightly better labeled by ML. Human experts rated both types of descriptions as similarly valuable, although both types show decreased value in the B criteria vs. the A criteria.

By focusing ML algorithms on labeling individual behaviors and not on labeling the final case label, our approach supports transparent clinical decision-making without replacing it. Our data and analysis may inspire future diagnostic adjustments and the role of different criteria and data sources. Especially when further insights in the spectrum and different subgroups can be established, the value of the criteria may increase or decrease for different groups.

AI, both traditional (22, 23) and generative (22), has the potential to advance equity and innovation in healthcare. Significant challenges remain, including bias in data and deployment, limited availability of high-quality datasets, risks of new forms of errors, and substantial resource demands. Nonetheless, basic research projects such as ours demonstrate the potential to contribute to more accessible and accurate screening and diagnosis, thereby helping shape a more just and effective future for AI-enabled care.

## Data Availability

The datasets presented in this article are not readily available because the data used to train the ML algorithms and to evaluate the diagnostic tests are not publicly available due to the ADDM Network Data Confidentiality and Security Agreement and the EHRs' limitation of data to university researchers. The parameters used to train the ML algorithm and AI prompts are described in the article. Requests to access the datasets should be directed to gondyleroy@arizona.edu.
