# Supplementary material for: Utility of lay and clinical narratives for transparent autism diagnosis using BioBERT deep learning
Source: Front Digit Health. 2026 Jul 17;8:1804334. doi: 10.3389/fdgth.2026.1804334 (PMC13424195; doi:10.3389/fdgth.2026.1804334)
Supplement: Supplementary file 1 [file Supplementaryfile1.docx]

**Appendix A: BioBERT Model parameters**

The following parameters were used for both the lay and clinical examples:

- The learning rate was set at 1 x 10 -5 = 0.00001 and a sigmoid activation function was used. Training (first dataset) and tuning (second dataset) differed in their learning rate, as is commonly done, so that the first trained model is not overpowered by subsequent learning. Model fine-tuning was conducted with a reduced learning rate of 5 × 10^-7.
- Early stopping was used to prevent overfitting using monitored validation loss. If the validation loss didn’t improve by a minimum amount (minimum delta = 0.007) for four consecutive epochs (patience), we stopped training.
- Loss function: binary cross-entropy loss function.
- Optimizer: We have used the create_optimizer function from the transformer’s library, where the default optimizer is ADAM. The number of warm-up steps at 20% of the training steps (length of train dataset for each fold * number of epochs), i.e., the number of training steps during which the learning rate is gradually increased from zero to its initial value.

The lay text data model was trained on the University of Arizona’s High Performance Computing (HPC) system, utilizing 32 GB of RAM, 4 CPUs, and one P100 GPU. The training duration per epoch remained consistent, averaging approximately 40 minutes to 2 hours, with total training time ranging from 60 to 200 hours per model.

The clinical text data model was trained in a Paperspace (cloud) environment equipped with 45 GB of RAM, 8 CPUs, and a 24 GB GPU. Each epoch required approximately 1 hour on average, resulting in a total training time of 70 to 80 hours per model.

Due to different computational time opportunities and limitations, the lay and clinical text data models differed in the total epochs used, which were 10 for the lay and 8 for the clinical text data, with a batch size set to 8 for the lay and 16 for the clinical model. However, for consistency, both models used the same parameters for early stopping, so the models were not allowed to overfit beyond the point of best validation performance.

**Appendix B: Prompt to Summarize Behavioral Examples:**

### CONTEXT

You are an expert clinician reading clusters of ASD-related clinical observations.

### OBJECTIVE

Generate a short, clinician-facing **title** that summarises the main findings of THIS cluster only.

### STYLE

Plain clinical English; no jargon beyond common ASD terminology.

### TONE

Professional and objective.

### AUDIENCE

Pediatricians evaluating a child for autism; the title appears in an EHR summary list.

### RESPONSE REQUIREMENTS

• ONE sentence, **maximum 20 words**.

• Include at least **one concrete behavior or test result** that is prominent in the example (e.g., “inconsistent name response”, “delayed joint attention”).

• Use ONLY facts found in the examples; **do NOT invent or generalise**.

• No lists, bullets, numbering, or boilerplate like “assessment of”.\n"

• Remove names, dates, MRNs; no placeholder tokens (<n>, </n>, <sep_*).

### EXAMPLES

*Examples followed here.*

**Appendix C**

Table 6: Two-tailed Pearson Correlations between Sample Size and Performance for the Seven Diagnostic Criteria (N=7)

|  |  | **Lay**  **r (p-value)** | **Clinical**  **r (p-value)** | **Combined**  **r (p-value)** |
| --- | --- | --- | --- | --- |
| **Lay** | | | | |
|  | **Precision** | -.186 (.689) |  |  |
|  | **Recall** | .560 (.191) |  |  |
| **Clinical** | | | | |
|  | **Precision** |  | -.271 (.556) |  |
|  | **Recall** |  | .561 (.190) |  |
| **Combined** | | | | |
|  | **Precision** |  |  | .148 (.752) |
|  | **Recall** |  |  | .077 (.870) |

**Appendix D**

Table 7: Scores for Lay and Clinical Examples for Utility, Specificity, Clinical Representativeness, and Impact on Daily Life with Independent Samples T-Test (two-tailed) conducted for the Source (Lay and Clinical)

| **Source** | **DSM**  **Criterion** | **Utility** | **Specificity** | **Clinical Rep.** | **Impact Daily Life** | **Avg** |
| --- | --- | --- | --- | --- | --- | --- |
| **Lay** | | | | | |  |
|  | A1 | 3.7 | 3.1 | 3.8 | 3.6 | 3.6 |
|  | A2 | 3.7 | 3.1 | 3.8 | 3.6 | 3.6 |
|  | A3 | 3.4 | 3.3 | 3.5 | 3.5 | 3.4 |
|  | B1 | 3.4 | 3.0 | 3.4 | 2.8 | 3.2 |
|  | B2 | 3.0 | 3.0 | 2.9 | 2.9 | 3.0 |
|  | B3 | 2.4 | 2.3 | 2.6 | 2.6 | 2.5 |
|  | B4 | 2.8 | 2.1 | 2.3 | 2.1 | 2.3 |
|  | **Avg** | **3.20** | **2.84** | **3.18** | **3.01** | **3.1** |
| **Clinical** | | | | | |  |
|  | A1 | 3.6 | 3.4 | 3.9 | 3.8 | 3.7 |
|  | A2 | 3.6 | 3.4 | 3.9 | 3.8 | 3.7 |
|  | A3 | 3.0 | 2.9 | 3.3 | 3.5 | 3.2 |
|  | B1 | 2.8 | 2.7 | 3.3 | 3.2 | 3.0 |
|  | B2 | 2.7 | 2.5 | 3.0 | 3.0 | 2.8 |
|  | B3 | 2.3 | 2.4 | 2.6 | 2.6 | 2.5 |
|  | B4 | 2.4 | 2.0 | 2.4 | 2.4 | 2.3 |
|  | **Avg** | **2.91** | **2.756** | **3.20** | **3.18** | **3.0** |
| **p-value** |  | .311 | .748 | .964 | .581 |  |

Table 8: Scores for Lay and Clinical AI-generated Title Summaries for Concise with Independent Samples T-Test (two-tailed) conducted for the Source (Lay and Clinical)

| **Source** | **Criterion** | **Concise** | **Rep. Examples** | **Avg.** |
| --- | --- | --- | --- | --- |
| **Lay** | | | |  |
|  | A1 | 3.0 | 3.6 | 3.3 |
|  | A2 | 3.0 | 3.6 | 3.3 |
|  | A3 | 2.7 | 2.8 | 2.8 |
|  | B1 | 2.6 | 3.0 | 2.8 |
|  | B2 | 2.6 | 3.3 | 3.0 |
|  | B3 | 2.1 | 2.3 | 2.2 |
|  | B4 | 2.3 | 2.4 | 2.4 |
|  | **Avg.** | **2.61** | **3.00** | **2.8** |
| **Clinical** | | | |  |
|  | A1 | 3.4 | 3.1 | 3.3 |
|  | A2 | 3.4 | 3.1 | 3.3 |
|  | A3 | 2.4 | 2.6 | 2.5 |
|  | B1 | 2.1 | 2.6 | 2.4 |
|  | B2 | 2.1 | 2.4 | 2.3 |
|  | B3 | 1.9 | 2.2 | 2.1 |
|  | B4 | 1.9 | 2.1 | 2.0 |
|  | **Avg** | **2.46** | **2.58** | **2.5** |
| P-value |  | .587 | .125 |  |

**Appendix E**

Table 9: Correlation analysis between expert scores for clinical data (Two-tailed Pearson correlation coefficient).

|  | **Expert 1** | | | | | |
| --- | --- | --- | --- | --- | --- | --- |
| **r (p-value)** | **Concise Examples** | **Representative Examples** | **Utility** | **Specificity** | **Clinical Representativeness** | **Impact on Daily Life** |
| Expert 2 |  |  |  |  |  |  |
| **Concise Examples**  **(N=103)** | .278 (.004) |  |  |  |  |  |
| **Representative Examples**  **(N=103)** |  | .425 (.000) |  |  |  |  |
| **Utility**  **(N=105)** |  |  | .504 (.000) |  |  |  |
| **Specificity**  **(N=105)** |  |  |  | .292 (.003) |  |  |
| **Clinical Representativeness**  **(N=105)** |  |  |  |  | .548 (.000) |  |
| **Impact on Daily Life**  **(N=105)** |  |  |  |  |  | .628 (.000) |

Table 10: Correlation analysis between expert scores for lay data (Two-tailed Pearson correlation coefficient).

|  | **Expert 1** | | | | | |
| --- | --- | --- | --- | --- | --- | --- |
| **r (p-value)** | **Concise Examples** | **Representative Examples** | **Utility** | **Specificity** | **Clinical Representativeness** | **Impact on Daily Life** |
| **Expert 2** |  |  |  |  |  |  |
| **Concise Examples**  **(N=103)** | .324 (.058) |  |  |  |  |  |
| **Representative Examples**  **(N=103)** |  | .473 (.004) |  |  |  |  |
| **Utility**  **(N=105)** |  |  | .334 (.050) |  |  |  |
| **Specificity**  **(N=105)** |  |  |  | .370 (.029) |  |  |
| **Clinical Representativeness**  **(N=105)** |  |  |  |  | .690 (.000) |  |
| **Impact on Daily Life**  **(N=105)** |  |  |  |  |  | .673 (.000) |
